# Transcriptomic Analysis of Liquid Non-Sporulating *Streptomyces coelicolor* Cultures Demonstrates the Existence of a Complex Differentiation Comparable to That Occurring in Solid Sporulating Cultures

**DOI:** 10.1371/journal.pone.0086296

**Published:** 2014-01-21

**Authors:** Paula Yagüe, Antonio Rodríguez-García, María Teresa López-García, Beatriz Rioseras, Juan Francisco Martín, Jesús Sánchez, Angel Manteca

**Affiliations:** 1 Área de Microbiología, Departamento de Biología Funcional and IUOPA, Facultad de Medicina, Universidad de Oviedo, Oviedo, Spain; 2 Instituto de Biotecnología de León, INBIOTEC, Parque Científico de León, León, Spain; Queen’s University Belfast, United Kingdom

## Abstract

*Streptomyces* species produce many clinically relevant secondary metabolites and exhibit a complex development that includes hyphal differentiation and sporulation in solid cultures. Industrial fermentations are usually performed in liquid cultures, conditions in which *Streptomyces* strains generally do not sporulate, and it was traditionally assumed that no differentiation took place. The aim of this work was to compare the transcriptomes of *S. coelicolor* growing in liquid and solid cultures, deepening the knowledge of *Streptomyces* differentiation. Microarrays demonstrated that gene expression in liquid and solid cultures were comparable and data indicated that physiological differentiation was similar for both conditions. Eighty-six percent of all transcripts showed similar abundances in liquid and solid cultures, such as those involved in the biosynthesis of actinorhodin (*actVA, actII-4*) and undecylprodigiosin (*redF*); activation of secondary metabolism (*absR1, ndsA*); genes regulating hydrophobic cover formation (aerial mycelium) (*bldB*, *bldC*, *bldM*, *bldN*, *sapA, chpC*, *chpD*, *chpE*, *chpH*, *ramA, ramC*, *ramS*); and even some genes regulating early stages of sporulation (*wblA, whiG, whiH*, *whiJ*). The two most important differences between transcriptomes from liquid and solid cultures were: first, genes related to secondary metabolite biosynthesis (CDA, CPK, coelichelin, desferrioxamine clusters) were highly up-regulated in liquid but not in solid cultures; and second, genes involved in the final stages of hydrophobic cover/spore maturation (*chpF, rdlA*, *whiE, sfr)* were up-regulated in solid but not in liquid cultures. New information was also provided for several non-characterized genes differentially expressed in liquid and solid cultures which might be regulating, at least in part, the metabolic and developmental differences observed between liquid and solid cultures.

## Introduction

**Figure 1:**
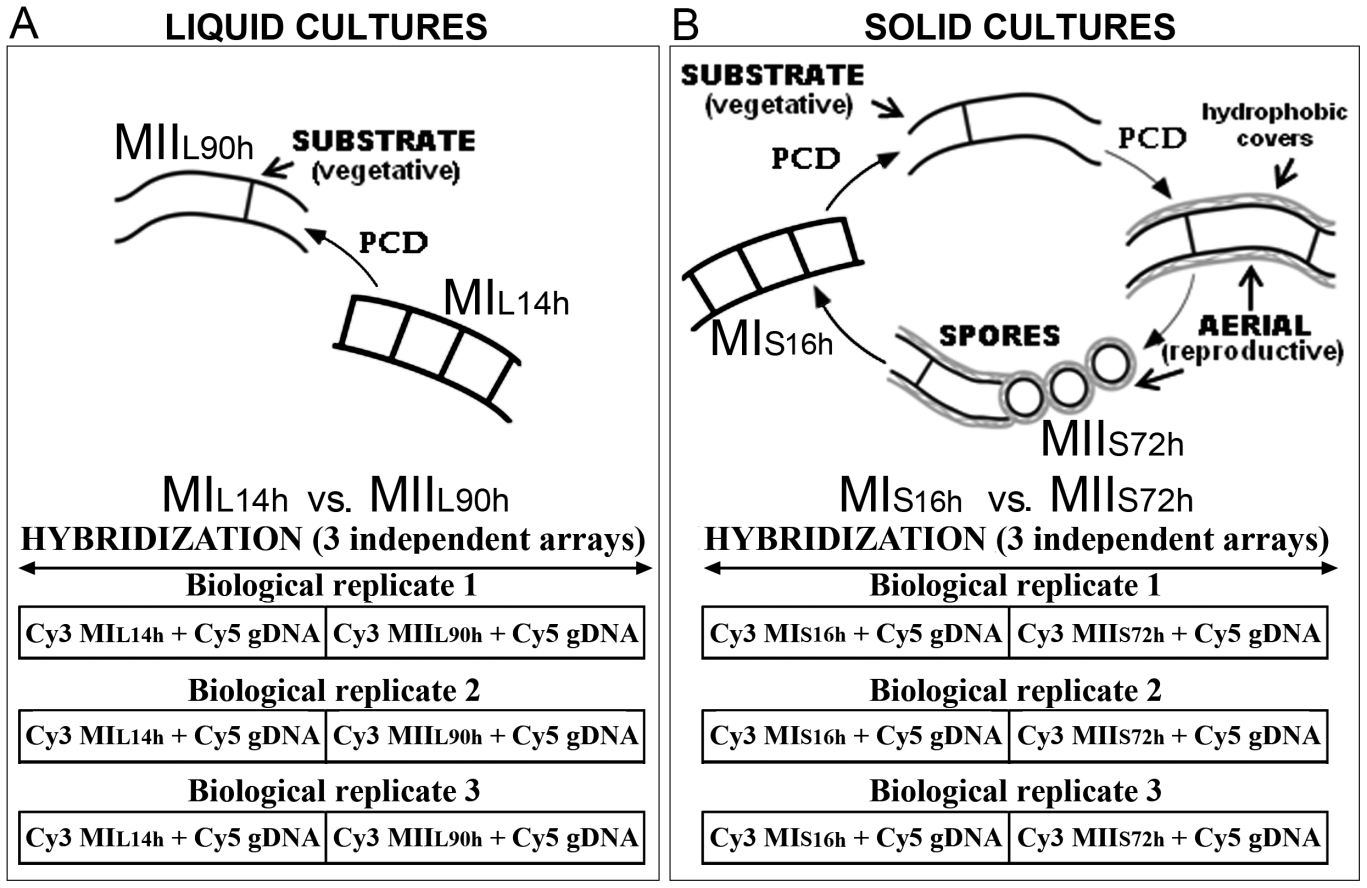
*Streptomyces coelicolor* development stages and sample preparation. (A) Liquid non-sporulating cultures. (B) Solid sporulating cultures. Mycelial structures (MI, first compartmentalized mycelium; MII, second multinucleated mycelium). The classical nomenclature of substrate, aerial mycelium, and hydrophobic layers are indicated. Three independent biological replicates and two developmental stages (MI and MII) were processed and cDNA was labeled with Cy3 while chromosomal *S. coelicolor* DNA was used as reference and labeled with Cy5. The scheme was adapted from Manteca et al. [12].

Approximately two-thirds of the clinical antibiotics, as well as a large number of eukaryotic cell differentiation inducers and inhibitors are synthesized by members of the *Streptomyces* genus [1]–[4]. Streptomycetes undergo a complex developmental cycle, which includes sporulation in solid cultures. Industrial processes for secondary metabolite production are performed in liquid cultures (large bioreactors), conditions in which most streptomycetes do not sporulate and it was generally assumed that differentiation processes were absent under these conditions [5]–[9]. During the last few years, new insights concerning *Streptomyces* differentiation during pre-sporulation stages were discovered in solid and liquid cultures. After spore germination, a compartmentalized mycelium (MI) initiates development and the MI compartments are separated by membranous septa which generally do not display thick cell walls (reviewed in [10]). A fraction of MI cells undergo a highly-ordered programmed cell death (PCD) [11] and the remaining viable cells differentiate into a multinucleated mycelium with sporadic septa (MII). It is the MII stage which produces antibiotics and sporulates on solid culture medium ([8], outlined in Fig. 1).

Proteomic analyses demonstrate that differentiation in liquid non-sporulating cultures was more similar to sporulating cultures on solid medium than expected in the context of the classical *Streptomyces* developmental model focusing on hydrophobic cover formation and sporulation stages [12]. This work further extends upon studies analyzing gene expression during development.

There are several *Streptomyces* transcriptomic studies describing genetic expression at different developmental time points in liquid cultures [13], [14], at particular time points in solid cultures [15], [16], and comparing *Streptomyces* mutants with the wild type strain in liquid [17] or solid [18], [19] cultures. However, in our knowledge this study is the first to specifically compare differences between *S. coelicolor* liquid and solid cultures. Knowledge about the genes differentially expressed in liquid and solid cultures will contribute to understanding the biochemical pathways regulating pre-sporulation developmental stages in *Streptomyces* and the activation of secondary metabolism.

## Materials and Methods

### 2.1. Bacterial Strains and Media

*Streptomyces coelicolor* M145 was used in this study. In order to facilitate data comparison with previous morphological [8] and proteomic [12] studies, the same culture conditions from those works were used: liquid cultures were performed in sucrose free R5A liquid media where 20 ml of culture medium were inoculated directly with spores (1×10^7^ spores per ml) into flasks of 100 ml and incubated at 200 rpm and 30°C.

### 2.2. Sampling of *Streptomyces coelicolor* Cells throughout the Differentiation Cycle

*S. coelicolor* grown in liquid culture were processed at different developmental time points (14 and 90 hours) (outlined in Fig. 1). The 14-hour time point corresponded to the first compartmentalized mycelium (MI_L_) and 90 hours to the second multinucleated mycelium (MII_L_). Three independent cultures were prepared and processed (biological replicates). Samples (20 ml from 14 h culture, 2 ml from 90 h culture) were centrifuged at 1000 g (5 minutes at 2°C) and the cells preserved in RNA Protect (Qiagen®) at −80°C.

### 2.3. RNA Isolation and Microarray Hybridization

Total RNA was isolated from 3 biological replicates using phenol extraction and the *RNeasy Midi Kit* (Qiagen). RNA integrity was verified using the 2100 Bioanalyzer (Agilent). cDNA samples were synthesized and labeled using random hexamers, *SuperScript* III reverse transcriptase (Invitrogen), and Cy3-dCTP (GE Healthcare Life Sciences). Remnant RNA was hydrolyzed with NaOH and retrotranscription products were purified using the *MinElute PCR Purification Kit* (Qiagen). Genomic DNA from *S. coelicolor* M145 was used as the common reference. gDNA was labeled with Cy5-dCTP (GE Healthcare Life Sciences) using the *BioPrime Array CGH Genomic Labeling Module* (Invitrogen), purified with the *MinElute* kit, and labeling efficiencies quantified with a *NanoDrop* ND-1000 spectrophotometer. Mixtures of Cy3-cDNA (825 ng)/Cy5-gDNA (20 pmol of Cy5) were prepared in 110 µl of hybridization buffer (1 M NaCl, 100 mM MES, pH 6.5, 20% formamide, 20 mM EDTA, 1% Triton X-100). The microarrays used for gene expression analysis were obtained from Oxford Gene Technology in the 4×44k format (Agilent *ink-jet* technology) comprised of 4 identical matrices of 43,798 experimentally-validated probes (60-mer oligonucleotides) covering ORFs and intergenic regions of the *S. coelicolor* genome [18]. The hybridization mixes (100 µl) were applied to the microarray surface following the manufacturer’s instructions, and hybridized at 55°C for 67 hours. The slide was washed in 50 ml of Agilent *Gene Expression Wash Buffer 1* for 5 min at room temperature and then in 50 ml of *Wash Buffer 2* preheated to 37°C for 1 min, both with horizontal agitation (85 rpm). The slide was then briefly immersed in the Agilent *Stabilization and Drying Solution* prior to measuring fluorescence with an Agilent DNA Microarray Scanner G2565BA using the extended dynamic mode. Quantification of fluorescence intensities was performed using the *FeatureExtraction* software (v9.5.1, Agilent).

### 2.4. Transcriptome Data Analysis

Fluorescence intensities were processed using the R environment (R Development Core Team 2011, version 2.12.2) and the limma package [20]. For each spot on the microarray, net fluorescence intensities were calculated subtracting the median of background pixels values from the mean of foreground pixels. When the intensity value was negative or lower than the background standard deviation, the standard deviation of the background pixels was used as the surrogate intensity, while *S. coelicolor* genomic DNA labeled with Cy5 was used as the common reference. The Mg values were calculated as the log2 of the Cy3-cDNA intensity divided by the Cy5-gDNA intensity and were normalized using probe weights by first cyclic loess (window of 0.3, 3 iterations) and then global median.

BLAST comparisons indicated that 943 of the array probes had potential cross-hybridization with more than one gene and that 7234 of the probes corresponded to intergenic regions. Weight values of 10^−6^ were assigned to these non-valid probes and a weight value of 1 assigned to the valid probes (35621). Values from valid probes of the same gene were averaged and limma linear models were used to obtain the log2 abundance values of comparisons between two conditions and the associated *p*-values, both FDR-corrected and uncorrected for multiple testing. The complete array data (Mg and *p*-values) for the 7721 transcripts quantified in this work are included in Table S1.

### 2.5. Computational and Bioinformatics Analyses

For comparison of relative transcript abundance values between MI and MII, the MI stage was used as the reference, and abundance values were shown as the log2 ratio of MII/MI. Positive abundances corresponded to transcripts up-regulated in MII, negative abundance values to transcripts up-regulated in MI and abundance values were considered significant when they were higher than 1 (2-fold up-regulation in MII) or lower than −1 (0.5-fold up-regulation in MII). Differences between liquid and solid cultures were considered significant when their coefficients of variation were higher than 0.7 (Table 1 and Table 2). ProteinCenter 2.0 (Proxeon, Denmark) was used to analyze and process genes. Genes were classified into functional categories according to their annotated functions in the Gene Bank database, and by homology/functions according to the Gene Ontology, Conserved Domain, KEGG pathway and StrepDB databases.

**Table 1:** Transcripts showing opposite (positive values in solid and negative in liquid or *vice versa*) and significant (coefficient of variation between solid and liquid cultures higher than 0.7) abundances (Fig. 4C, F, I, L, O, R, U; Fig. 5C, F, I, L).

Function	SCO no.	Description	Log 2 (MII/MI)		SCO no.	Description	Log 2 (MII/MI)	
			S	L			S	L
**PRIMARY** **METABOLISM**	SCO0992	Cysteine synthase	−1.1	1.2	SCO3909	50S ribosomal protein L9, *rplI*	−1.2	0.3
	SCO1132	Oxidoreductase	−0.4	1.8	SCO4152	5′-nucleotidase	0.7	−1.1
	SCO1134	Oxidoreductase	−1.2	1.9	SCO4284	Deacetylase	−0.5	1.1
	SCO1181	Putative plasmid partition protein	−2.2	1.2	SCO4577	Helicase DEAD-like	−1.2	1.1
	SCO1335	Oxidoreductase	−1.7	0.2	SCO4701	30S ribosomal protein S10	1.2	−1.5
	SCO1343	Uracil-DNA glycosylase	−1.2	0.3	SCO4702	50S ribosomal protein L3	0.5	−2
	SCO1600	Translation initiation factor IF-3	1.2	−0.9	SCO4703	50S ribosomal protein L4	0.4	−2.1
	SCO1631	Helicase	−1.2	0.8	SCO4712	50S ribosomal protein L14	0.7	−1.4
	SCO1966	Excinuclease ABC subunit B	−1.9	0.1	SCO4734	50S ribosomal protein L13	0.6	−1.8
	SCO2003	DNA polymerase I	−1.8	0.2	SCO5494	NAD-dependent DNA ligase *ligA*	−2.2	0.9
	SCO2036	Tryptophan synthase subunit alpha	−0.4	1.4	SCO5566	ATP-dependent DNA helicase *recG*	−1.2	0.1
	SCO2597	50S ribosomal protein L21	0.9	−1.4	SCO5815	ATP-dependent DNA helicase	−1.4	0.7
	SCO2770	Agmatinase. Urea cycle and metabolism of amino groups	0.4	−1.1	SCO5920	DEAD-box RNA helicase	−1.7	0
	SCO3351	DNA repair protein RadA	−1.6	0.2	SCO6084	DNA polymerase III subunit epsilon	−2.6	0.8
	SCO3434	DNA polymerase I	−1	0.8	SCO6262	Helicase	−1.6	0
	SCO3543	Putative DNA topoisomerase I	1.2	−0.2	SCO6662	Transaldolase	−3.8	0
**SECONDARY** **METABOLISM**	SCO0490	Esterase	−1.1	1.4	SCO3243	Myo-inositol phosphate synthase	−0,6	1.6
	SCO0491	ABC transporter	−0.8	1	SCO6273	Type I polyketide synthase *cpkC*	−1.3	2.5
	SCO0492	Peptide synthetase	−1.1	1.2	SCO6274	Type I polyketide synthase *cpkB*	−1.9	3.2
	SCO0493	ABC-transporter	−1	1.7	SCO6275	Type I polyketide synthase *cpkA*	−1.9	3.2
	SCO0497	Iron-siderophore permease	−1.9	0.9	SCO6276	Secreted monooxygenase	−2.3	7.9
	SCO0498	Peptide monooxygenase	−2.3	2.7	SCO6277	Epoxide hydrolase	−2.3	6
	SCO0499	Methionyl-tRNA formyltransferase	−1.6	1.9	SCO6278	Membrane transport protein	−2	5.2
	SCO2782	Pyridoxal-dependent decarboxylase	−3.2	0.4	SCO6279	Aminotransferase	−1.9	6.4
	SCO3229	4-hydroxyphenylpyruvic dioxygenase	−1	1.6	SCO6280	Putative transcriptional regulator	−0.7	4.8
	SCO3235	ABC transporter	−1	1.8	SCO6281	FAD-binding protein	−0.6	4.3
**DIFFERENTIA-TION**	SCO2705	ChpF	1.4	−0.4	SCO5112	BldKA	1	−2.2
	SCO2607	Sfr protein; sporulation protein	0.6	−1.3	SCO5114	BldKC	0.6	−2.4
	SCO4069	SarA	−0.3	1.6	SCO5190	DNA-binding protein, *wblC*	3.2	−1.6
	SCO4823	Possible target for *bldA* regulation	1	−2.8				
**REGULATORY**	SCO0646	TetR family transcriptional regulator	−1.2	0.1	SCO4303	Transcriptional regulator	−2.2	0.3
	SCO1568	TetR family transcriptional regulator	−1.8	2.4	SCO4336	MarR-family protein	−2.6	0.2
	SCO2489	TetR family transcriptional regulator	−2	1	SCO4375	MarR family regulatory protein	−1.6	0.1
	SCO2845	GntR family transcriptional regulator	−1.3	0.1	SCO4413	AraC transcription regulator similar to S griseus AdpA	−1	1.9
	SCO2958	Transcriptional regulator, *nnaR*	−1	0.2	SCO5607	Transcriptional regulator	−0.5	1.8
	SCO3066	Putative regulator of Sig15	−0.5	1	SCO6267	Putative transcriptional regulator	−1.3	1.8
	SCO3167	TetR family transcriptional regulator	−3.1	0.6	SCO6268	Histidine kinase	−2.4	2.5
	SCO3275	merR family transcriptional regulator	0.4	−1.2	SCO6565	Transcriptional regulator	−1.4	0
	SCO3390	Two component sensor kinase	−1.7	1	SCO6694	Transcriptional regulator	−0.9	2.5
	SCO3696	Transcriptional regulator	−1.1	0.6	SCO6743	Transcriptional accessory protein	−0.9	1.2
	SCO3848	Serine/threonine protein kinase	1	−0.3	SCO7364	TetR transcriptional regulator	−2.3	0.4
	SCO3879	DnaA replication initiation protein	0.6	−1.4	SCO7585	MerR transcriptional regulator	−0.6	2
	SCO4005	RNA polymerase sigma factor	−2.1	3.5	SCO7694	TetR transcriptional regulator	−0.8	1
	SCO4145	Polyphosphate kinase, *ppk*	−1.3	0.5				
**TRASPOSONS -IS**	SCO7074	Transposase	1.4	−0.2				
**CONJUGATION-RECOMBINATION-MUTAGENESIS**	SCO5102	MutT-like protein	−0.6	1.6	SCO5770	Recombination regulator, *recX*	−1.3	3.3
	SCO5769	Recombinase A, *rec A*	−1.3	2.2				
**STRESSS - DEFENSE**	SCO0774	Cytochrome P450	−2.9	1.7	SCO3890	Thioredoxin reductase	−1.3	0.1
	SCO0180	Usp, universal stress protein family	−1.1	1.5	SCO4761	Co-chaperonin GroES	−1.8	1.5
	SCO3669	Chaperone protein DnaJ	−0.5	1.6	SCO4762	GroEL1	−1.4	0.5
	SCO3670	Heat shock protein GrpE	−0.8	1.6	SCO5254	SodN, superoxide dismutase	−1.2	1.3
	SCO3701	Putative thioredoxin reductase	−1.7	0.6				
**CATABOLISM -DEGRADATION**	SCO3000	Phosphatase	−1.2	1.2	SCO4798	Peptidase	1.1	−0.5
	SCO3487	Agarase	3.8	−1	SCO5285	ATP-dependent protease	−1.3	3.6
	SCO3661	ATP-dependent protease, *clpB*	−2.1	0.7	SCO5446	Probable zinc metalloprotease	1.6	0
	SCO3712	Hydrolase	−1.9	1.1	SCO6109	Probable secreted hydrolase	−1.4	0.1
	SCO4241	Proteinase	1.2	−0.3	SCO7263	Chitinase	1.2	−0.2
**TRANSPORTERS - SECRETED**	SCO0119	Possible small secreted protein	1	−0.3	SCO3607	Secreted protein	−4.1	0.1
	SCO0994	Putative transport permease protein	−0.7	2	SCO4243	Putative secreted protein	0.7	−1.5
	SCO0286	Putative peptidoglycan binding protein	0.5	−2.8	SCO4289	Possible secreted protein	−1.9	0.1
	SCO1292	Putative secreted protein	−1.6	1.3	SCO4585	ABC transporter protein	−0.3	2.4
	SCO1515	Preprotein translocase subunit SecF	0.4	−1.2	SCO4722	Preprotein translocase SecY	0.5	−1.5
	SCO1516	Preprotein translocase subunit SecD	0.5	−1.5	SCO4993	Metal ion transport protein	0.8	−1.1
	SCO1567	Transmembrane-transport protein pqrB	−1.4	2.3	SCO5016	Possible secreted protein	1.1	−0.3
	SCO1785	Iron-siderophore	−1.3	0.1	SCO5130	ABC transporter	0.6	−1.3
	SCO2074	Lipoprotein signal peptidase	0.7	−1.3	SCO6199	Possible secreted esterase	−1.3	2.1
	SCO2949	Carboxyvinyltransferase, murA	0.5	−1.2	SCO6272	Possible secreted protein	−1.9	1.9
	SCO3024	Transport protein	0.5	−1.3	SCO6320	Transport membrane protein	−1.1	0.1
	SCO3166	Membrane transport protein	−2.3	0.6	SCO6665	Probable secreted glucosidase	0.4	−1
	SCO3286	Putative secreted protin	−3.1	0.5	SCO6980	ABC transporter membrane protein	1.4	0
	SCO3366	Exporter	−1.8	0.4	SCO7453	Putative secreted protein	3.4	−0.6

Average log2 abundance values from three biological replicates of the MII with respect to MI in solid (S) and liquid (L) cultures. Only transcripts with significant abundances are shown (log2 abundance greater than ±1 in liquid and/or solid cultures). Functions (according to Gene bank, Gene Ontology, Conserved Domain, and KEGG and StrepDB databases): Primary metabolism (DNA/RNA replication, aerobic and anaerobic energy production, glycolysis and glyconeogenesis, pentose phosphate pathway, amino acid metabolism, nucleotide metabolism, translation, protein folding, RNA/protein processing, nucleases/RM methylases); secondary metabolism (secondary metabolites synthesis); differentiation (TTA *bldA* targets, Bld and Whi proteins); regulatory genes (transcriptional regulators, kinases, other regulatory genes); transposons - insertion sequences; conjugation – recombination - mutagenesis; stress and defense proteins; catabolism - degradation; lipid metabolism; transporters and secreted (ABC transporters, transporters and secreted proteins). Genes with “unknown” functions (Table S1) were not included.

**Table 2:** Transcripts up- or down- regulated in MI or MII liquid and solid cultures, showing differences in their abundances (coefficient of variation between liquid and solid higher than 0.7).

Function	SCO no.	Description	Log 2 (MII/MI)		SCO no.	Description	Log 2 (MII/MI)	
			S	L			S	L
**PRIMARY** **METABOLISM**	SCO0922	Succinate dehydrogenase	−2.3	−0.5	SCO4631	Type IV restriction endonuclease	−3	−0.7
	SCO1321	Elongation factor Tu	1.4	0	SCO4685	DEAD-box RNA helicase	−1.1	0
	SCO1522	Glutamine amidotransferase	−1.8	−0.6	SCO5059	Polyphosphate glucokinase, ppgK	−2	−0.4
	SCO1679	Gluconokinase	−2.8	−0.5	SCO5657	Aldehyde dehydrogenase	−1.4	0
	SCO2470	Deoxyguanosinetriphosphate Triphosphohydrolase-like protein	−1.7	−0.2	SCO6211	Uricase	2.1	0.4
	SCO2655	Putative nuclease	0.9	3.9	SCO6341	Exonuclease	−2	−0.3
	SCO3023	S-adenosyl-L-homocysteine hydrolase	1.7	0	SCO6415	Probable dihydropyrimidinase	1.1	0.3
	SCO3303	Lysyl-tRNA synthetase	−0.4	−1.4	SCO6661	Glucose-6-phosphate dehydrogenase	−2.7	−0.4
	SCO3542	Putative thymidine kinase	−1	−0.3	SCO6769	Aminotransferase	0.3	1.6
	SCO4606	NADH dehydrogenase subunit NuoL2	−0.3	−1.3	SCO6962	Glutamine synthetase	0.9	3.2
	SCO4608	NADH dehydrogenase subunit NuoN2	−0.5	−2.1	SCO7511	Glyceraldehyde 3-phosphate dehydrogenase, gap2	−2.7	−0.7
**SECONDARY** **METABOLISM**	SCO0188	Methylesterase	−1.3	−0.4	SCO5693	Acyl CoA dehydrogenase	1.4	0.5
	SCO0190	Methyltransferase	−2.4	−0.7	SCO6286	ScbR2	1.8	6.6
	SCO1267	Acyl carrier protein	−3.9	0	SCO6750	Isopentenyl-diphosphate isomerase	0.6	2.8
	SCO2478	Reductase activated by actinorhodin	0.2	4.2	SCO6760	Phytoene synthase	0.4	1.2
**DIFFERENTIA-TION**	SCO2718	RdlA	7.4	0.9	SCO5316	WhiE	4	0.1
	SCO4346	Possible target for *bldA* regulation	−1.5	−0.1				
**REGULATORY**	SCO0193	Putative transcriptional regulator	−2.5	−0.4	SCO5006	Septum site-determining protein	0.4	1.3
	SCO0447	MarR family regulatory protein	−1.5	−0.5	SCO5008	Putative septum site-determining protein	0.6	2
	SCO0767	Putative transcriptional regulator	0.3	1.3	SCO5264	Putative transcriptional regulator	−1.7	−0.2
	SCO1034	TetR family transcriptional regulator	0.2	1.9	SCO5540	Histidine kinase-like ATPases	1.1	0.3
	SCO1801	Two component response regulator	0.5	2.6	SCO5616	Putative transcriptional regulator	−1.6	−0.5
	SCO2223	TetR family transcriptional regulator	−0.5	−1.7	SCO5656	Transcriptional regulatory protein	−3.8	−0.5
	SCO2253	Putative transcriptional regulator	1.2	0.1	SCO5785	Transcriptional regulator	2	0.5
	SCO2730	Putative TetR- transcriptional regulator	−1.9	−0.4	SCO6154	Putative transcriptional regulator	0.6	2
	SCO2879	Putative MinC septum formation inhibitor	0.4	1.7	SCO6219	Ser/Thr protein kinase	−2.2	−0.5
	SCO3367	TetR family transcriptional regulator	−1.2	−0.2	SCO6424	Putative histidine kinase	2.3	0.7
	SCO4019	GntR family regulatory protein	−1.4	−0.3	SCO6566	ROK family protein	1.8	0.2
	SCO4021	Two component system histidine kinase	−0.5	−1.9	SCO6696	Regulatory protein	0.2	1.6
	SCO4032	MarR regulatory protein	−5.4	−0.2	SCO6778	Transcriptional regulator	−1.2	−0.3
	SCO4223	AraC family transcription regulator	−1.4	−0.1	SCO7014	LacI transcriptional regulator	−4.5	−0.8
	SCO4261	LuxR regulatory protein	−3.6	−0.3	SCO7016	LacI transcriptional regulator	−2.1	−0.6
	SCO4308	Transcriptional regulator	−1.4	−0.2	SCO7061	MarR transcriptional regulator	−1.3	−0.2
	SCO4640	TetR transcriptional regulator	−4.3	−0.2				
**TRASPOSONS -IS**	SCO7740	Insertion element	1.5	0				
**CONJUGATION-RECOMBINATION-MUTAGENESIS**	SCO7442	Putative conjugal transfer protein TrbL	−2	0				
**STRESSS - DEFENSE**	SCO0885	Thioredoxin	0.5	1.8	SCO4609	Putative heat shock peptidase *htpX*	−1.5	−0.4
								
**CATABOLISM -DEGRADATION**	SCO0591	Putative lysozyme	2.7	0.2	SCO6078	Probable alpha amylase	0.3	1.4
	SCO0740	Probable hydrolase	−0.9	−2.8	SCO6324	Putative hydrolase	2.2	0.4
	SCO1509	Possible hydrolase	−0.5	−1.6	SCO6414	Possible hydrolase	1.2	0.4
	SCO1643	20S proteasome alpha-subunit	0.5	2	SCO7205	Putative hydrolase	−1.7	0
	SCO3779	Probable nucleoside hydrolase	−0.3	−1.4	SCO7473	Phenylacetic acid degradation protein PaaC	0.7	3.3
	SCO4108	Probable peptidase	1.8	0.5	SCO7474	Phenylacetic acid degradation protein PaaD	0.4	1.5
	SCO4288	Possible phosphatase	−1.4	−0.5	SCO7590	Catalase	1.6	0.2
	SCO5456	Putative glycosyl hydrolase	0.3	1.7				
**TRANSPORTERS - SECRETED**	SCO0796	Putative membrane transporter	−0.3	−1.4	SCO3507	Integral membrane efflux protein	−0.3	−1.3
	SCO1044	Possible secreted protein	−2	−0.2	SCO3513	Possible secreted protein	−1.5	−0.2
	SCO1144	ABC transporter ATP-binding protein	−1.9	−0.5	SCO3718	Potassium-transporting ATPase	−0.3	−2.1
	SCO1147	ABC transporter	−0.5	−2	SCO4031	Membrane transport protein	−3	−0.5
	SCO1822	Transmembrane transport protein	1.6	0.4	SCO4424	Possible secreted protein	−1	−0.2
	SCO2010	ABC transporter Branched chain amino acid transport permease	1.1	0.3	SCO4471	Possible secreted protein	−1.9	−0.6
	SCO2011	Putative amino acid transport	0.5	2.2	SCO4641	Transmembrane efflux protein	−2.5	−0.5
	SCO2383	Possible secreted protein	−1.1	−0.3	SCO6086	Transport system integral membrane protein	−1.9	−0.1
	SCO2756	Predicted permease	−0.5	−1.4	SCO6104	Possible secreted protein	0.7	3.2
	SCO2780	Secreted protein	−2	−0.3	SCO6258	Sugar ABC transporter permease	−0.4	−1.4
	SCO2905	Putative sugar permease	−1.5	−0.2	SCO6417	Integral membrane transporter	1.5	0.3
	SCO3402	Possible secreted protein	−1.1	−0.1				

(Fig. 4B, E, H, K, N, Q, T; Fig. 5B, E, H, K). Average log2 abundance values from three biological replicates of MII with respect to MI in solid (S) and liquid (L) cultures. Only transcripts with significant abundances are shown (log2 abundance greater than ±1 in liquid and/or solid cultures). Functions as in Table 1. Genes with “unknown” functions (Table S1) were not included.

## Results and Discussion

### 3.1. Global Quantification of Gene Transcriptions in Liquid Cultures

In order to facilitate comparison with previous work [16], MI and MII transcriptomes were analyzed in liquid non-sporulating cultures using a workflow similar to that used for solid sporulating cultures [16]: three independent biological replicates and two developmental stages (MI and MII) were processed (outlined in Fig. 1). MI from liquid cultures (MIL) was obtained at early time points (14 hours), long before the onset of the PCD and differentiation of MII, and MII (MIIL) was obtained at 90 hours long after the disappearance of the MI [8] (Fig. 1A). The transcriptomes from both developmental stages were compared with those of solid cultures (MIS and MIIS) [16] (Fig. 1B).

**Figure 2:**
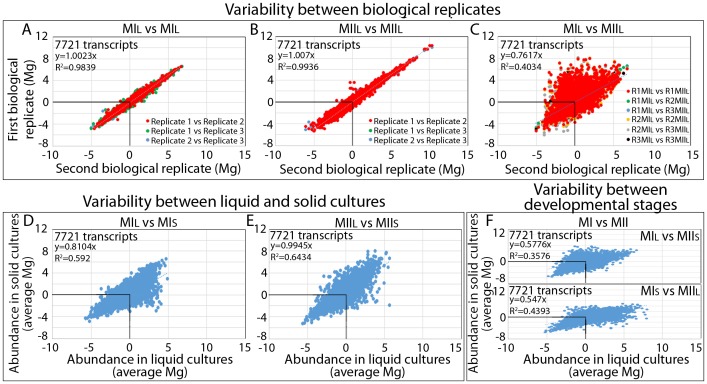
Quantitative transcriptomic data analysis. Correlation of transcription abundance values (log2 ratio against chromosomal DNA). Upper panels - biological replicates: (A) MIL vs. MIL, (B) MIIL vs. MIIL, (C) MIL vs. MIIL (three biological replicates compared in pairs). Lower panels - developmental stages (average abundance values from three biological replicates: (D) MIL vs. MIS, (E) MIIL vs. MIIS, (F) MIL vs. MIIS and MIS vs. MIIL.

The relative abundances of 7721 transcripts were estimated and normalized to chromosomal DNA (see full Mg data in Table S1). Reproducibility between biological replicates of MIL and MIIL (Fig. 2A, B) was high (regression coefficients of 0.98 and 0.99 respectively) and much higher than correlation between abundance values from different developmental stages (MIL *vs.* MIIL; regression coefficient of 0.4) (Fig. 2C). Correlation between the Mg values from liquid cultures obtained in this work (average from the three biological replicates) and the Mg values reported for sporulating solid cultures [16] was also high for MIL *vs.* MIS (Fig. 2D) and MIIL *vs.* MIIS (Fig. 2E) (regression coefficient of 0.6 in both cases). Correlation between different developmental stages (MI *vs.* MII) in solid and liquid cultures (Fig. 2F) was comparable to the correlation observed between different developmental stages in liquid cultures (regression coefficients of 0.4 in all cases) (compare Fig. 2C with Fig. 2F).

**Figure 3:**
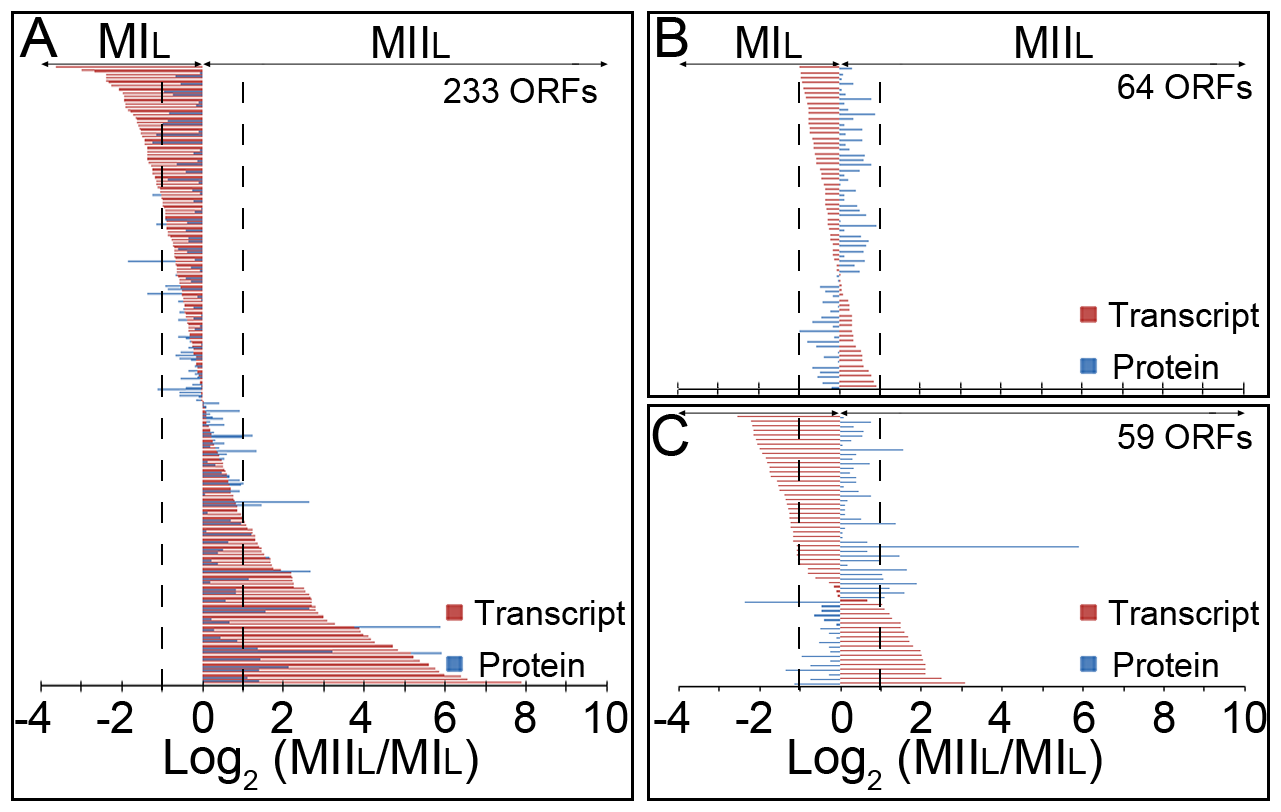
Comparison between transcriptomics (this work) and proteomics [12]. Transcription and protein abundance values correspond to log2 MIIL/MIL and are the average of two (in the case of proteomics) or three (in the case of transcriptomics) biological replicates. Dashed lines indicate the limit for considering abundance variations as significant (log2 abundances greater than ±1). (A) Proteins and transcripts significantly up-regulated in MIIL (positive abundance values higher than 1) or in MIL (negative abundance values lower than −1). (B) Proteins and transcripts without significant variations (log 2 abundances within ±1 interval). (C) Proteins and transcripts showing divergent abundance values (positive values in MIL and negative in MIIL or vice versa).

The abundances of MI and MII transcripts (this work) were compared with the MI and MII protein abundances previously reported for liquid cultures [12] using the MI stage as reference, and both, transcript/protein abundances were shown as the log2 ratio of MII/MI. Three hundred fifty-six of the proteins encoded by the 7721 transcripts identified in this work have been previously quantified by proteomics [12]. Two hundred thirty-three proteins and transcripts were significantly up-regulated in MII (positive abundance values higher than 1) or MI (negative abundance values lower than −1) (Fig. 3A); 64 did not differ significantly (log 2 abundances within ±1 interval) (Fig. 3B); and 59 ORFs showed divergent abundances (positive protein abundance and negative transcript abundance or *vice versa*) (Fig. 3C). Overall, correlation between protein and transcript abundance was reasonable, considering they are different biomolecules with different kinetics and turnover rates.

### 3.2. Similarities and Differences between MI and MII Transcriptomes in Solid and Liquid Cultures

**Figure 4:**
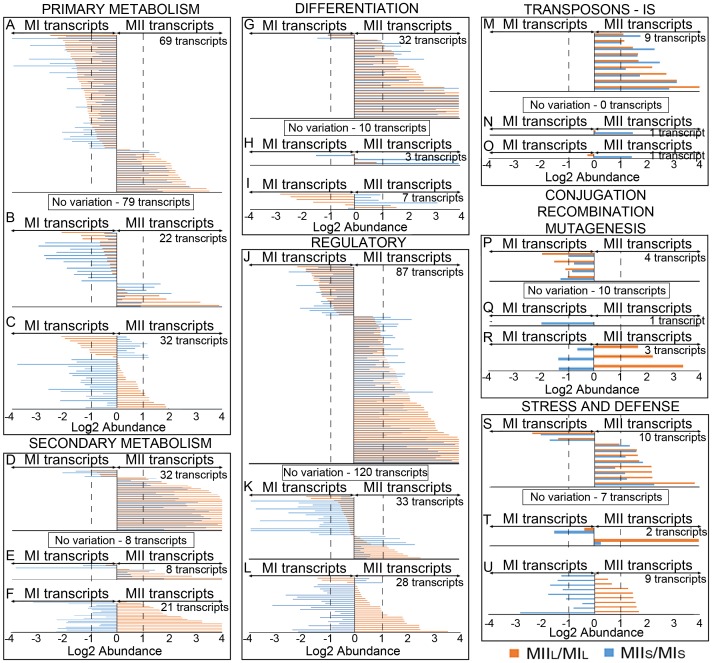
Abundance values of transcripts quantified in solid and liquid cultures (1901 in total) and grouped into functional categories. Abundance values (average of log2 MII/MI from three biological replicates) and transcripts with significant variations in MIIL and/or MIIS (log2 abundance greater than ±1) are shown. Transcripts without significant (log2 abundance within the ±1interval) are indicated into squares and labeled as “No variation”. Functional categories: primary metabolism (DNA/RNA replication, aerobic and anaerobic energy production, glycolysis and glyconeogenesis, pentose phosphate pathway, amino acid metabolism, nucleotide metabolism, translation, protein folding, RNA/protein processing, nucleases/RM methylases); secondary metabolism (secondary metabolite synthesis); differentiation (TTA *bldA* targets, Bld and Whi proteins); regulatory genes (transcriptional regulators, kinases, other regulatory genes); transposons - insertion sequences; conjugation, recombination, mutagenesis; stress and defense proteins. Dashed lines indicate the limit for considering abundance variations significant (log2 abundance ±1).

**Figure 5:**
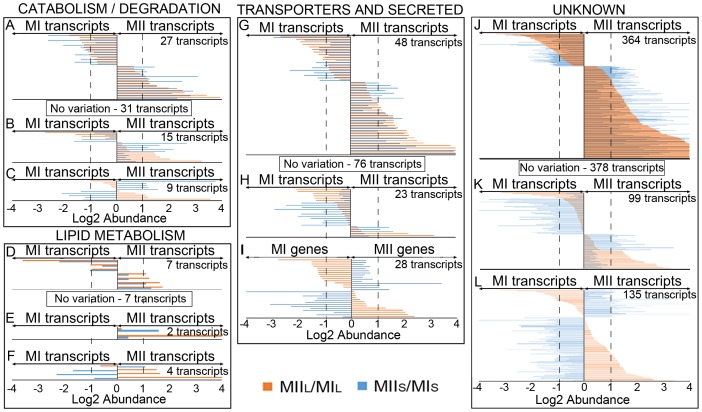
Transcripts abundance values (log2 MII/MI) grouped in functional categories (continuation). Functional categories: catabolism and degradation; lipid metabolism; transporters and secreted (ABC transporters, transporters and secreted proteins); genes with unknown function. Dashed lines indicate the limit for considering abundance variations significant (log2 abundance ±1).

MI and MII transcriptomes from liquid (this work) and solid cultures [16] were compared, using the MI stage as the reference, and showing transcript abundances as the log2 ratio of MII/MI (Fig. 4, Fig. 5) (Table S1). When transcript abundances with very high confidence, described by Yagüe et al [16] in solid cultures (1901 transcripts), were compared to the abundances detected in liquid cultures (this work), it was evident that transcriptomes of MI and MII were very similar for both conditions (Fig. 4, Fig. 5): 1420 transcripts (75% of the total) were up-regulated (positive abundance values higher than 1), down-regulated (negative abundance values lower than −1) in MII with respect to MI, or were not significantly different (log 2 abundances within ±1 interval) (Fig. 4A, D, G, J, M, P, S; Fig. 5A, D, G, J); 204 transcripts (11% of the total) were up- or down- regulated in MI or MII in liquid and solid cultures but showed important differences in their abundance (coefficient of variations between liquid and solid abundances higher than 0.7) (Fig. 4B, E, H, K, N, Q, T; Fig. 5B, E, H, K) while 277 (14% of the total) showed significantly (coefficient of variation between liquid and solid cultures higher than 0.7) opposite (positive values in solid and negative in liquid or *vice versa*) abundances (Fig. 4C, F, I, L, O, R, U; Fig. 5C, F, I, L).

#### 3.2.1. Transcripts showing opposite abundances in solid and liquid cultures

The most important differences between liquid and solid cultures were for the 277 transcripts showing opposite (positive values in solid and negative in liquid or vice versa) and significant abundances (coefficient of variation between solid and liquid cultures higher than 0.7) (Table 1). These transcripts included 21 genes encoding for proteins involved in “primary metabolism” whose biological roles in development remain unknown. Interestingly, one third of these 21 genes encode for ribosomal proteins, indicating that translational machinery may play a relevant role in controlling parts of *Streptomyces* differentiation, as has been previously suggested [21].

The 20 genes involved in “secondary metabolism” which had opposite differences in liquid and solid cultures (Table 1), were up-regulated in MIIL (up to 239-fold), and down-regulated in MIIS (up-to 0.1-fold) with respect to MI. These included genes from calcium-dependent antibiotic (CDA), yellow antibacterial pigment (Cpk), and coelichelin clusters [22] as well as SCO2782, one of the genes putatively involved in desferrioxamine biosynthesis [22]. These results indicated production of these secondary metabolites in liquid but not in solid cultures, and illustrated that in addition to differentiation (development of MII), there were specific regulatory mechanisms for the production of different secondary metabolites under different culture conditions.

Six of the 7 genes related to hyphal “differentiation” differentially expressed in liquid and solid cultures were up-regulated in MII from solid sporulating cultures, and down-regulated in MII from non-sporulating liquid cultures (Table 1) and included genes related to aerial mycelium/spore hydrophobic cover formation (*chpF*, *sfr*, *bldkA*, *bldkC*) [23]. Also included were *wblC*, one of the *whiB*-like regulatory genes highly conserved in *Streptomyces*, whose biological function remains to be characterized [24] and SCO4823, encoding for a putative protein with a TTA leucine codon which converts it to a putative target for *bldA* [25]. *SarA* was the only “differentiation” transcript up-regulated in MIIL (3-fold) and down-regulated in MIIS (0.8-fold). Interestingly, *sarA* was conserved in the *Streptomyces* genus and it is known to repress sporulation and activate antibiotic production in solid cultures [26]. Repression of sporulation and increased antibiotic production in liquid cultures are the most important phenotypic differences between MIIL and MIIS, and *sarA* may be one of the master genes regulating this process.

Twenty-four of the 27 “regulatory” genes differentially expressed in liquid and solid cultures (Table 1) were up-regulated in MIIL (up to 11-fold) and down-regulated in MIIS (up to 0.18-fold). These genes included *pqrA*, a TetR-like transcriptional regulator involved in resistance to oxidative stress [27]; *nnaR*, a transcriptional regulator which appears to be involved in the regulation of nitrate/nitrite assimilation [28]; SCO4005, a sigma factor which is activated by the stringent factor ppGpp [29]; and *ppk* (SCO4145) which represses antibiotic production [30]. The biological functions of the other 21 “regulatory” genes up-regulated in MIIL have yet to be characterized. Only 3 “regulatory” genes were up-regulated in MIIS (up to 2-fold) and down-regulated in MIIL (up to 0.4-fold) (Table 1): SCO3275, a putative MerR transcriptional regulator of unknown function; SCO3848, a putative serine/threonine protein kinase of unknown function and *dnaA* a chromosomal replication initiator protein [31]. Up-regulation of *dnaA* in MIIS may be involved with the division of chromosomal DNA during sporulation.

Other genes differentially expressed in liquid and solid cultures were SCO7074, a putative transposase up-regulated in MIIS (2.6-fold), and genes related to “conjugation, recombination, or mutagenesis” such as *recA*, *recX* and SCO5102 (*mutT*-like), which were up-regulated in MIIL (up to 10-fold), and down-regulated in MIIS (up to 0.4-fold), which suggested activation of mechanisms of genetic variability in liquid but not in solid cultures.

All transcripts encoding for “stress and defense” proteins (9 genes) were up-regulated in MIIL (up to 3.2-fold), and down-regulated in MIIS (up to 0.1-fold), which suggested the existence of greater stress in liquid cultures than in solid cultures. These transcripts included well-characterized genes such as chaperones (*dnaJ*, *grpE*, *groES*, *groEL1*), superoxide dismutases (*sodN*), and thioredoxin reductases (SCO3890).

Ten genes encoding for proteins related to “catabolism and degradation” were also differentially expressed in liquid and solid, but their expression was not clearly biased toward MI or MII (Table 1). These transcripts included SCO3487, a gene encoding for an agarase [32], which was up regulated in MIIS (14-fold) but down-regulated in MIIL (0.5-fold), and could be involved in agar degradation in solid cultures. The biological significance of the remainder of these catabolic MI or MII genes in liquid and solid cultures, is yet to be characterized.

Twenty-eight transcripts encoding for “transporters and secreted proteins” were differentially expressed in liquid and solid cultures (Table 1) though the biological role of these genes in controlling development remains unknown. Interestingly *pqrB*, encoding for a transmembrane-transport protein, was co-expressed with SCO1568 (a TetR transcriptional regulator, discussed above), and both were up-regulated in MIIL (up to 5-fold) and are known to play a role in oxidative stress [27].

#### 3.2.2. Transcripts up- or down- regulated in MI or MII in liquid and solid cultures showing significant differences in their abundances

Two hundred and nine transcripts were up- or down- regulated in MI or MII for liquid and solid cultures and showed important differences in abundance (coefficient of variation between liquid and solid higher than 0.7) (Fig. 4B, E, H, K, N, Q, T; Fig. 5B, E, H, K) (Table 2). These genes, together with the genes described in the previous paragraph (Table 1), were potentially involved, in the regulation of the developmental/metabolic differences between liquid and solid cultures. Of these, 24 transcripts were involved in “primary metabolism” (Table 2): genes for oxidative phosphorylation, TCA cycle, glycolysis, glyconeogenesis, biosynthesis of amino acids, RNA translation, etc. Eight genes putatively involved in “secondary metabolism” also showed significant differences between liquid and solid cultures (Table 2). SCO2478, a reductase induced by actinorhodin [33], SCO6750, an isopentenyl-diphosphate isomerase putatively involved in terpenoid biosynthesis (sco00900 KEGG pathway), SCO6286 or *scbR2*, a pseudo-γ-butyrolactone receptor that represses the SARP regulator *kasO* of the yellow antibacterial pigment (Cpk) cluster [34], and SCO6760, a putative phytoene synthase [22], were highly up-regulated in MII with respect to MI in liquid cultures (18-, 7-, 97- and 2.3-fold respectively) but not in solid (1.1-, 1.5-, 3.5- and 1.3-fold respectively). The opposite was seen for SCO5693, a putative acyl CoA dehydrogenase involved in the biosynthesis of secondary metabolites (the sco01110 KEGG pathway), which was overexpressed 2.6-fold in MII with respect to MI in solid cultures, but only 1.4-fold in liquid. The other 3 genes encoding for proteins putatively involved in “secondary metabolism” were down-regulated in MII liquid and solid cultures, and were the only exceptions to secondary metabolite genes up-regulated in MI: two genes putatively involved in isorenieratene biosynthesis (SCO0188 and SCO0190); and SCO1267, one of the genes encoding for an acyl carrier protein [22]. These genes may not be activated under the culture conditions used in this work.

Two key “differentiation” genes were also differentially expressed in liquid and solid cultures (Table 2). *RdlA*, one of the genes involved in the last stages of aerial mycelium/spore hydrophobic cover maturation [35] was up-regulated 168-fold for MII in solid cultures with respect to MI, but only 1.8-fold in liquid cultures. *WhiE*, a gene responsible for the biosynthesis of an aromatic polyketide precursor to the gray spore pigment [36] was up-regulated 16-fold for MII in solid cultures but expressed at the same level in MI and MII liquid cultures.

Two of the “regulatory” genes differentially expressed in liquid and solid cultures had already been characterized (Table 2): SCO4223, an AraC transcriptional regulator involved in resistance to oxidative stress [37] up-regulated in MI liquid cultures (2.6-fold *vs.* 1-fold in solid) and SCO5785, a transcriptional regulator which enhances antibiotic production [38] up-regulated in MII solid cultures (4-fold *vs.* 1.4-fold in solid). The biological functions of the other 31 “regulatory” transcripts differentially expressed in liquid and solid cultures remain unknown (Table 2).

One putative insertion element (SCO7740) was up-regulated in MII with respect to MI in solid cultures (2.8-fold), but not in liquid (1-fold, no variation) and a putative conjugal transfer protein (SCO7442) was up-regulated in MI liquid (4-fold) but not solid cultures (1-fold, no variation) (Table 2). Two genes related to “stress and defense” (SCO0885, SCO4609) were differentially expressed in liquid and solid cultures (Table 2). SCO0885 encoded for a thioredoxin which is induced under oxidative stress [29] and was especially up-regulated for MII in liquid (3.5-fold vs. 1.4-fold in solid), suggesting the existence of more stress in liquid than in solid cultures.

For genes involved in “catabolism and degradation”, a putative lysozyme (SCO0591) was up-regulated in MII solid cultures (6.5-fold *vs.* 1.1-fold in liquid) and may play a role during the excision of individual spores. Twenty-three genes putatively encoding for “transporters and secreted” proteins (Table 2) and 95 genes encoding for “unknown” proteins (Fig. 5K. Table S1) were also differentially expressed in liquid and solid cultures, though further work will be necessary to characterize their biological function.

#### 3.2.3. Transcripts showing similar abundances in solid and liquid cultures

The 1420 transcripts with similar abundance in solid and liquid cultures (Table S1), included several well-characterized genes (summarized in Table 3). Genes involved in “primary metabolism” (Fig. 4A) were mostly up-regulated in MI: up to 18-fold in the case of oxidative phosphorylation and the TCA cycle genes, up to 4-fold in the case of genes encoding proteins involved in glycolysis and glyconeogenesis, and up to 3.7-fold in the case of genes encoding ribosomal proteins (Table 3).

**Table 3:** Summary of well characterized genes whose transcripts showed similar abundances in liquid and solid cultures (coefficient of variation between liquid and solid cultures lower than 0.7) (Fig. 4A, D, G, J, M, P, S; Fig. 5A, D, G, J).

Function	SCO no.	Description	Log 2 (MII/MI)		SCO no.	Description	Log 2 (MII/MI)	
			S	L			S	L
**PRIMARY** **METABOLISM**	SCO1947	Glyceraldehyde-3-phosphate dehydrogenase	−2	−2	SCO3945	Cytochrome oxidase CydA	−4.2	−2.2
	SCO2972	PrfB	−0.6	−1	SCO3946	Cytochrome oxidase CydB	−3.4	−1.7
	SCO3425	30S ribosomal protein S18	−0.8	−1.9	SCO4607	NADH dehydrogenase NuoM2	−0.7	−1.8
**SECONDARY** **METABOLISM**	SCO5077	ActVA	1.3	2.7	SCO5898	RedF	1.7	1.7
	SCO5085	ActII-4	2.6	5.9	SCO6992	AbsR1	5.8	5.8
								
**DIFFERENTIA-TION**	SCO0409	SapA	2.7	3.4	SCO4768	BldM	3.5	4.5
	SCO1674	ChpC	4.3	4.5	SCO5582	NdsA	3.1	6.2
	SCO1675	ChpH	4.7	4.9	SCO5621	WhiG	0.7	1.7
	SCO1800	ChpE	5.3	7.2	SCO5723	BldB	1	2.6
	SCO2717	ChpD	5.6	3.4	SCO5819	WhiH	1.8	1.5
	SCO3323	BldN	4.2	4.8	SCO6681	RamC	1	0.9
	SCO3579	WblA	3.5	6.7	SCO6682	RamS	4.6	5.8
	SCO4091	BldC	1.4	2.7	SCO6683	RamA	0.9	0.7
	SCO4543	WhiJ	−1.4	−0.9				
**REGULATORY**	SCO0155	TetR family transcriptional regulator	−2.1	−0.8	SCO2232	Maltose operon repressor	−0.8	−1
	SCO1193	TetR family transcriptional regulator	−1.3	−0.7	SCO4034	Sigma factor sigN	1.3	2.8
	SCO1626	Cytochrome P450, rarE	2.4	3.9	SCO4180	Iron uptake regulatory protein	−0.7	−1.3
	SCO1628	RarC homologue	2.7	4.3	SCO4377	Serine-threonine kinase, afsL	−1.2	−1
	SCO1629	RarB homologue	2.8	4.6	SCO4850	TetR transcriptional regulator	−1.1	−0.6
	SCO1630	RarA homologue	3.7	5.8	SCO5820	hrdB sigma factor	1.7	1
	SCO2077	DivIVA	0.9	2.2	SCO7809	TetR transcriptional regulator	−2.2	−1.3
**TRASPOSONS -IS**	SCO2236	Plasmid maintenance killer protein	2.3	1.5	SCO6208	Putative Transposase	1.2	2.2
	SCO2311	Putative transposes	2.5	1.7	SCO6393	Transposase	1.6	1.7
	SCO3714	Transposase	3.1	3.1	SCO6394	IS element ATP binding protein	1.7	1.1
	SCO4350	Integrase	1.7	2.8	SCO6395	Putative IS element transposase	1	1.1
	SCO4772	Transposase	2.9	4.7				
**CONJUGATION-RECOMBINATION-MUTAGENESIS**	SCO1520	Holliday junction resolvase ruvC	−0.7	−1.5	SCO3876	Recombination protein F	−0.8	−1.1
								
**STRESSS – DEFENSE**	SCO0379	Catalase, katA	1.2	2.2	SCO5803	SOS regulatory protein LexA	2.3	3.8
	SCO0560	Catalase/peroxidase cpeB	1.2	2.2				
**CATABOLISM -DEGRADATION**	SCO5444	Possible glycogen phosphorylase, glgP	2.1	4.1				
**TRANSPORTERS – SECRETED**	SCO2008	Branched chain amino acid binding protein	1.9	2.8				

Average log2 abundance values (from three biological replicates) for the MII with respect to MI in solid (S) and liquid (L) cultures. Only transcripts with significant abundances are shown (log2 abundance greater than ±1 in liquid and/or solid cultures). Functions as in Table 1. Genes with “unknown” functions (Table S1) were not included.

Most genes involved in “secondary metabolism” (28 of 32) were up-regulated in MII (Fig. 4D): up to 60-fold in the case of genes from the actinorhodin cluster, 4.3-fold in the case of *redF*, a gene belonging to the prodigiosin cluster, and 56-fold in the case of *absR1*, a well-known activator of secondary metabolism [39].

Most of the well-characterized genes that participated in hyphal “differentiation” (30 of 32) were up-regulated in MII (Fig. 4G): up to 28-fold for activators of aerial mycelium differentiation (*bldB*, *bldC*, *bldN*, *bldM*); up to 147-fold for transcripts involved in the formation of hydrophobic covers (*sapA*, *chpC, chpD, chpE, chpH, ramA, ramC, ramS*); up to 3.2-fold for sporulation regulatory genes (*wblA*, *whiG*, *whiH*); and 73-fold for *ndsA*, a gene affecting antibiotic production [40]. *WhiJ*, a repressor of sporulation [41], was the only exception, as it was up-regulated during the non-sporulating phase (MI).

Interestingly, the expression of most “regulatory” genes (65 of 87) (Fig. 4J) was up-regulated in MII: up to 55-fold for “restoration of aerial mycelium formation” genes *rarA*-*C*, *rarE* homologues [42]; up to 4.6-fold for *DivIVA*, a gene essential for polar growth and morphogenesis [43]; and up to 7-fold for sigma factors *sigN* and *hrdB* (Table 3). The other 22 “regulatory” genes were up-regulated in MI, which included TetR transcriptional regulators, *afsL,* a serine-threonine kinase, as well as regulators involved in repressing maltose utilization (*malR*) [44] or regulation of nickel homeostasis/antioxidant response (*nur*) [45]. Several TetR family transcriptional regulators have been described in *Streptomyces* which function as repressors of antibiotic biosynthesis and export [46]–[48], so a role for these putative TetR transcriptional regulators in repressing the onset of antibiotic production in MI may be feasible; AfsL could be one of the proteins inactivating secondary metabolism in MI, as it has been shown to phosphorylate and regulate different regulators such as AfsR, a transcriptional activator involved in the regulation of secondary metabolism [49]; and the two regulators involved in repressing maltose utilization and nickel homeostasis/antioxidant response may be regulating metabolism of these compounds in MI and MII.

The expressions of the 9 genes encoding for putative “transposons and insertion sequences” which had similar abundances in liquid and solid cultures were up-regulated in MII (Fig. 4M) (Table 3), which suggested the activation of mobile genetic elements in solid and liquid MII cultures. The 4 transcripts involved in “conjugation, recombination, or mutagenesis” were up-regulated by as much 2.8-fold in MI (Fig. 4P) (Table S1) which included the well-characterized *recF* [50] and holliday junction resolvase *ruvC* [22] genes (Table 3), and may indicate the activation of DNA recombination in MI (prior to MII).

Most genes encoding for “stress and defense” proteins (8 of 10) were up-regulated up to 15-fold in MII (Fig. 4S). These transcripts included catalases, or the SOS regulatory protein LexA. The expression of genes related to “catabolism/degradation” (Fig. 5A), “lipid metabolism” (Fig. 5D), and “transport and secretion” (Fig. 5G), was not clearly biased toward MI or MII (Table S1). Some of these genes are well characterized, including *glgP*, a glycogen phosphorylase up-regulated in MII (up to 16-fold) which participates in glycogen and trehalose metabolism during sequential stages of aerial mycelium development [51] and SCO2008, encoding for a branched chain amino acid binding protein up-regulated in MII (up to 7-fold), which may participate in regulation of morphological differentiation in *S. coelicolor* [52]. Interestingly, most genes encoding for proteins of “unknown” function were up-regulated in MII (Fig. 5J).

## Conclusions and Future Perspectives

This work was the first specifically focused on comparing MI and MII transcriptomes from *S. coelicolor* liquid and solid cultures. Expression of most transcripts (86% of all the identified transcripts) was comparable between non-sporulating liquid cultures and sporulating solid cultures, including genes involved in the biosynthesis of actinorhodin (*actVA, actII-4*) and undecylprodigiosin (*redF*); certain activators of secondary metabolism (*absR1, ndsA*); genes regulating hydrophobic cover formation (aerial mycelium) (*bldB*, *bldC*, *bldM*, *bldN*, *sapA, chpC*, *chpD*, *chpE*, *chpH*, *ramA, ramC*, *ramS*) and a few genes regulating early stages of sporulation (*wblA, whiG, whiH*, *whiJ*). The important similarities between transcriptomes from liquid and solid cultures were especially relevant considering that MIIS was collected at the sporulation phase, and that different culture media were used for liquid and solid cultures. Consequently, the developmental stages (MI and MII) were comparable, independent of age (developmental time points) or culture conditions.

The two major important differences between transcriptomes from liquid and solid cultures were: first, the expression of genes related to secondary metabolite biosynthesis (CDA, CPK, coelichelin, desferrioxamine clusters) which were up-regulated in liquid but not solid cultures; and second, genes involved in the last stages of hydrophobic cover/spore maturation (*chpF, rdlA*, *whiE, sfr*), which were up-regulated in solid with respect to liquid cultures.

Overall, this work extended previous morphological and proteomic studies, demonstrating that differentiation in liquid non-sporulating cultures was more similar to solid sporulating cultures than expected based on hyphal morphology (aerial mycelium formation and sporulation), and concluded that physiological differentiation was similar under both culture conditions. New information was also provided for several un-characterized genes differentially expressed in liquid and solid cultures (Table 1 and Table S1), which may be regulating, at least in part, the metabolic and developmental differences observed in liquid and solid cultures. This study contributes to the knowledge needed to further understand the biochemical pathways controlling pre-sporulation developmental stages and the activation of secondary metabolism in *Streptomyces*.

## Supporting Information

Table S1 **Quantitative data for the expression of** ***Streptomyces coelicolor*** **transcripts.** The relative abundances of 7721 transcripts quantified in this work are shown as the log2 ratio of transcript abundance with respect to chromosomal DNA (Mg values) and to MI in both liquid (this work) and solid [16] cultures. Data are the average of three biological replicates and P-values are indicated. The 1901 transcripts quantified with very high confidence by Yagüe et al [16] in solid cultures (1901 transcripts) were compared with the transcripts obtained in this work for liquid cultures. Data were separated in four folders: three of them include data of Tables 1, 2 and 3; the other include data for transcripts without significant variations between solid and liquid cultures.
(XLSX)
